# Effects of fourteen essential minerals and vitamins on acute and chronic tubulointerstitial nephritis: a multivariate Mendelian randomization study

**DOI:** 10.1186/s41065-025-00383-x

**Published:** 2025-04-16

**Authors:** Xiaotan Pan, Zhiyan Guo, Yin Zheng, Cheng Su, Jiabo Chen

**Affiliations:** 1 Department of Pediatric Surgery, The First Affiliated Hospital of Guangxi Medical University, Nanning, Guangxi 530021 China; 2 Department of Rehabilitation Medicine, The First Affiliated Hospital of Guangxi Medical University, Nanning, Guangxi 530021 China; 3 Department of Pediatric Surgery, Maternity and Child Health Care of Guangxi Zhuang Autonomous Region, Nanning, Guangxi 530002 China

**Keywords:** Minerals, Vitamins, Tubulointerstitial nephritis, Mendelian randomization

## Abstract

**Objective:**

To investigate the causal relationship between minerals and vitamins and acute and chronic tubulointerstitial nephritis by Mendelian randomization.

**Methods:**

We selected fourteen minerals and vitamins from the GWAS database and acute tubulointerstitial nephritis and chronic tubulointerstitial nephritis from the Finnish database. Minerals and vitamins were first analyzed by two-sample Mendelian randomization for acute and chronic tubulointerstitial nephritis. The effects of minerals and vitamins on common acute and chronic tubulointerstitial nephritis were further explored by multivariate Mendelian randomization.

**Results:**

among fourteen minerals and vitamins by two-sample Mendelian randomization analysis, there was genetic causality for vitamin B6 and vitamin D on acute tubulointerstitial nephritis, and the results were vitamin B6 (β = -0.641; *P* = 0.049; OR = 0.527; 95% CI: 0.278–0.998); vitamin D (β = -3.165; *P* = 0.040; OR = 0.042; 95% CI: 0.002–0.861). Fourteen minerals and vitamins were not genetically causally associated with chronic tubulointerstitial nephritis. The presence of vitamin B6 was then analyzed by a multivariate Mendelian randomization study to independently affect acute tubulointerstitial nephritis and showed a negative correlation (*P* = 0.010; 95% CI: 0.021–0.159).

**Conclusion:**

We genetically predicted the possible influence of minerals and vitamins on acute and chronic tubulointerstitial nephritis. Vitamin B6 deficiency in vivo was found to adversely affect acute and chronic tubulointerstitial nephritis. This suggests that we pay clinical attention to the different effects that nutrients such as minerals and vitamins bring to acute and chronic tubulointerstitial nephritis.

**Clinical trial number:**

Not applicable.

## Introduction

Acute tubulointerstitial nephritis is a clinicopathologic syndrome in which renal interstitial inflammatory cell infiltration, interstitial edema, and renal tubular damage of varying degrees occur within a short period of time with renal insufficiency [1]. The disease is one of the more common causes of acute renal failure. Adverse drug reactions and infections are the most common causes of the disease. In addition, autoimmune diseases such as systemic lupus erythematosus, desiccation syndrome, transplant rejection, malignancy, metabolic, genetic, and physicochemical factors can also cause it. Most patients with acute tubulointerstitial nephritis have a good prognosis, while patients with severe pathologic damage or untimely treatment may be left with renal insufficiency and permanent renal impairment [2]. Chronic tubulointerstitial nephropathy is a renal interstitial disease characterized by chronic tubulointerstitial damage. Clinical manifestations include mild proteinuria, renal tubular dysfunction, and chronic renal failure [3]. The causes of chronic tubulo-interstitial nephritis mainly include hereditary diseases, infectious diseases, drug diseases, and systemic diseases. In clinical treatment, the main focus is to control and eliminate the cause of the disease and slow down the impairment of kidney function [4].

Minerals mainly include trace and macronutrients. In the human body, trace elements mainly include iron, copper, selenium and zinc, while macronutrients include calcium, potassium and magnesium [5]. Vitamins mainly contain carotene, folic acid, Vitamin B12, Vitamin B6, Vitamin C, Vitamin D, Vitamin E and so on [6]. The human body’s demand for minerals is very small, but it plays an irreplaceable role. Minerals act as activators of many enzymes and constitute important carriers in the body and are involved in the transfer of electrons in the body. Minerals are also involved in the synthesis of hormones and vitamins as well as influencing growth and development and the functioning of the immune system [7]. The vitamins required by the body are as important as the minerals and play similar roles. The roles of vitamins mainly include participation in important syntheses, metabolic reactions, and antioxidants in the body. Although the human body’s daily requirement of vitamins is very small, if the body’s long-term insufficient intake, it may lead to vitamin deficiencies, such as night blindness, rickets, anemia, scurvy and so on. In life, people should eat a reasonable diet and selectively supplement vitamins [8].

Tubulointerstitial nephritis, as a common kidney disease, is responsible for close to 20% of chronic kidney injury [9]. Tubulointerstitial nephritis’s bring a lot of irreversible damage to the kidney. At the same time, the clinical treatment is mainly symptomatic and lacks treatment and research on its etiologic mechanisms, especially from the genetic level. Therefore, this article takes nutrients as the starting point and selects minerals and vitamins as the factors that may affect the disease to analyze the effects of fourteen common minerals and vitamins required by the human body, including iron, copper, selenium, zinc, calcium, potassium, magnesium, carotenoids, folic acid, Vitamin B12, Vitamin B6, Vitamin C, Vitamin D, and Vitamin E, on the development of acute and chronic tubulointerstitial nephritis. Currently, research on minerals and vitamins and tubulointerstitial nephritis is limited to a few animal studies and case reports. In one study on animals, iron restriction was found to inhibit oxidative stress and inflammatory changes, contributing to increased protection against bovine serum albumin overload-induced renal tubulointerstitial injury in mice [10]. Another animal study found that iron deficiency did not affect the development of glomerular disease as determined by proteinurian one study on animals, iron restriction was found to inhibit oxidative stress and inflammatory changes, contributing to increased protection against bovine serum albumin overload-induced renal tubulointerstitial injury in mice. but was significant in preventing the development of tubulointerstitial disease and deterioration of renal function [11]. A case report demonstrates two patients with laboratory tests showing acute kidney injury and hypercalcemia and renal biopsies showing inflammatory interstitial nephritis and acute tubular necrosis. Treatment with furosemide and discontinuation of vitamins and anabolic substances resulted in recovery of renal function. The main causes of renal insufficiency were vitamin D toxicity and drug-induced interstitial nephritis [12]. Although these reports suggest an association between minerals and vitamins and tubular interstitial nephritis, the studies have only focused on the association between a single mineral or vitamin and tubular interstitial nephritis. This is due to experimental limitations or the presence of other confounding factors. Therefore, we envisioned the use of Mendelian randomized genome-wide association studies to explore the causal relationship between minerals and vitamins and tubulointerstitial nephritis at the genetic level. The research gap on the relationship between minerals and vitamins on renal tubulointerstitial nephritis can be bridged at the genetic level. At the same time, it also opens up new ideas for the treatment and prevention of tubulointerstitial nephritis from the perspective of minerals and vitamins in clinical practice.

Mendelian randomization analyzes the causal relationship between exposure factors and outcomes by introducing an instrumental variable as an intermediate variable. This method solves the problem that traditional experiments cannot effectively explain the causality between exposure factors and outcome variables due to confounding factors. Mendelian randomized genetic causality follows Mendelian laws of heredity, which states that if a genotype determines a phenotype, then the genotype can be associated with disease through that phenotype. In order to explore the relationship between phenotype and disease, and between disease and disease, Mendelian randomization studies are more effective. Therefore, the effects of 14 essential minerals and vitamins on acute and chronic tubular interstitial nephritis were investigated by Mendelian randomization. This method is a good way to study the relationship between the two at the genetic level.

## Methodology

### Research design

The Mendelian randomization study design follows Mendel’s laws of inheritance, where the genotype determines the phenotype and that genotype can be associated with disease through this phenotype. This genotype is used as an instrumental variable to study the relationship between phenotype and disease or disease and disease. It also avoids the influence of confounding factors in previous clinical studies.

The causal relationship between exposure factors fourteen minerals and vitamins required by the human body on the outcome factors of acute tubulointerstitial nephritis and chronic tubulointerstitial nephritis was first explored by two-sample Mendelian randomization separately. The results yielded a causal relationship between the presence of multiple minerals and vitamins among the fourteen minerals and vitamins required by the human body for acute tubulointerstitial nephritis. Finally, the minerals and vitamins most likely to influence acute tubulointerstitial nephritis were explored by multivariate Mendelian randomization.

GWAS summary information on fourteen minerals and vitamins required by the body and acute and chronic tubulointerstitial nephritis.

We collected fourteen minerals and vitamins required by the human body as exposure factors from the ieu Open Gaws Project (https://gwas.mrcieu.ac.uk/). The mineral and vitamin names and GWAS ID numbers are copper (ieu-a-1073), calcium (ukb-b-8951), zinc (ieu-a-1079), carotene (ukb-b-16202), folate (ukb-b-11349), iron (ukb-b-20447), magnesium (ukb-b-7372), potassium (ukb-b-17881), selenium (ieu-a-1077), Vitamin B12 (ukb-b-19524), Vitamin B6 (ukb-b-7864), Vitamin C (ukb-b-19390), Vitamin D (ukb-b-18593), Vitamin E (ukb-b-6888). All diseases were studied in individuals from Europe.

We selected acute tubulointerstitial nephritis and chronic tubulointerstitial nephritis as outcome factors from the Finnish database (https://r10.finngen.fi), respectively [13]. All diseases were studied in individuals from Europe. In Acute tubulointerstitial nephritis, the female population is 241,013 and the male population is 113,711. In chronic tubulointerstitial nephritis, the female population was 18,322 and the male population was 5172. The Acute tubulointerstitial nephritis consisted of 23,871 subjects and 21,306,078 SNPs. and the disease was defined using the N10 codes from the International Classification of Diseases, 10th edition (ICD-10). The chronic tubulointerstitial nephritis included 357,461 subjects and 21,305,468 SNPs, and was defined using ICD-10 N11 codes.

### Selection of genetic and instrumental variables

The instrumental variables selected for this study satisfy the three hypotheses of MR analysis: instrumental variables are associated with exposure factors; instrumental variables are not associated with confounders; and instrumental variables influence outcomes through exposure factors. We first obtained the relevant SNPs for fourteen minerals and vitamins required by the human body by genome-wide significance *p* < 5 × 10 − 8. Then we removed the chained imbalance between SNPs caused by strong LD by r2 < 0.001 and clumping distance = 10,000 kb. We then identified confounders associated with atherosclerosis, cerebral arterial occlusion, and stenosis by searching the literature. The PhenoScanner database was applied to exclude confounders. Next, palindromic SNPs with intermediate allele frequencies were deleted.Also, to ensure a stronger association of the instrumental variable with the exposure, we chose SNPs with an F-statistic > 10 as instrumental variables [14].The F-statistic was calculated using the formula F = beta^2^/se^215^.

#### GWAS relationship between fourteen minerals and vitamins required by the body for acute and chronic tubulointerstitial nephritis

Fourteen human-required minerals and vitamins acting causally in acute and chronic tubulointerstitial nephritis were obtained prior to the exclusion of palindromic SNPs and confounding SNPs, and ultimately, SNPs were used as IVs. to derive the correlation of independent genetic IVs with the GWAS of acute and chronic tubulointerstitial nephritis (Supplementary Material 1).

#### Tests of multiplicity and heterogeneity

Two-sample MR analyses between fourteen minerals and vitamins required by the human body and acute and chronic tubulointerstitial nephritis, respectively, were performed by the TwoSampleMR and MRPRESSO packages in R (version 4.3.1) [16].The MR Egger’s intercept test and the MR-PRESSO method were used to test for horizontal multivariate validity (*p* > 0.05), indicating that the genetic instrumental variables were not heterogeneous for the outcome factor [17]. No horizontal polytropy was detected in GWAS. The Cochran and Rucker Q statistics were used to detect heterogeneity in MR analysis, with *p* > 0.05 indicating no heterogeneity [18].

#### Mendelian randomization analysis and SNP effect analysis for the two samples

The mr_egger, weighted median, IVW, simple mode, and weighted mode methods were used to analyze the causal relationship between fourteen minerals and vitamins required by the human body and acute and chronic tubulointerstitial nephritis, respectively, and the results of IVW were used as the primary basis [19]. mr_egger, weighted median, simple mode, and weighted mode methods were used as the basis of auxiliary judgments [20]. p-values < 0.05 indicate that fourteen essential minerals and vitamins are causally associated with acute and chronic tubulointerstitial nephritis, respectively. “mr” and ‘mr_scatter_plot’ in R were used to verify the causal relationship between phenotype and disease [21]. The effect size of each SNP was determined using “mr_forest_plot” [22]. The sensitivity analysis “mr_leaveoneout_plot” was used to determine whether the relationship between phenotype and disease was affected by each SNP [23].

#### Multivariate mendelian randomization analysis

Multivariate Mendelian randomization analyses also used the results of the IVW as the primary basis. p-values < 0.05 indicate that excluding the effects between exposure factors still produced a causal effect on disease [24].

## Results

### Genetic causation of fourteen essential minerals and vitamins with acute tubulointerstitial nephritis

**Fig. 1:**
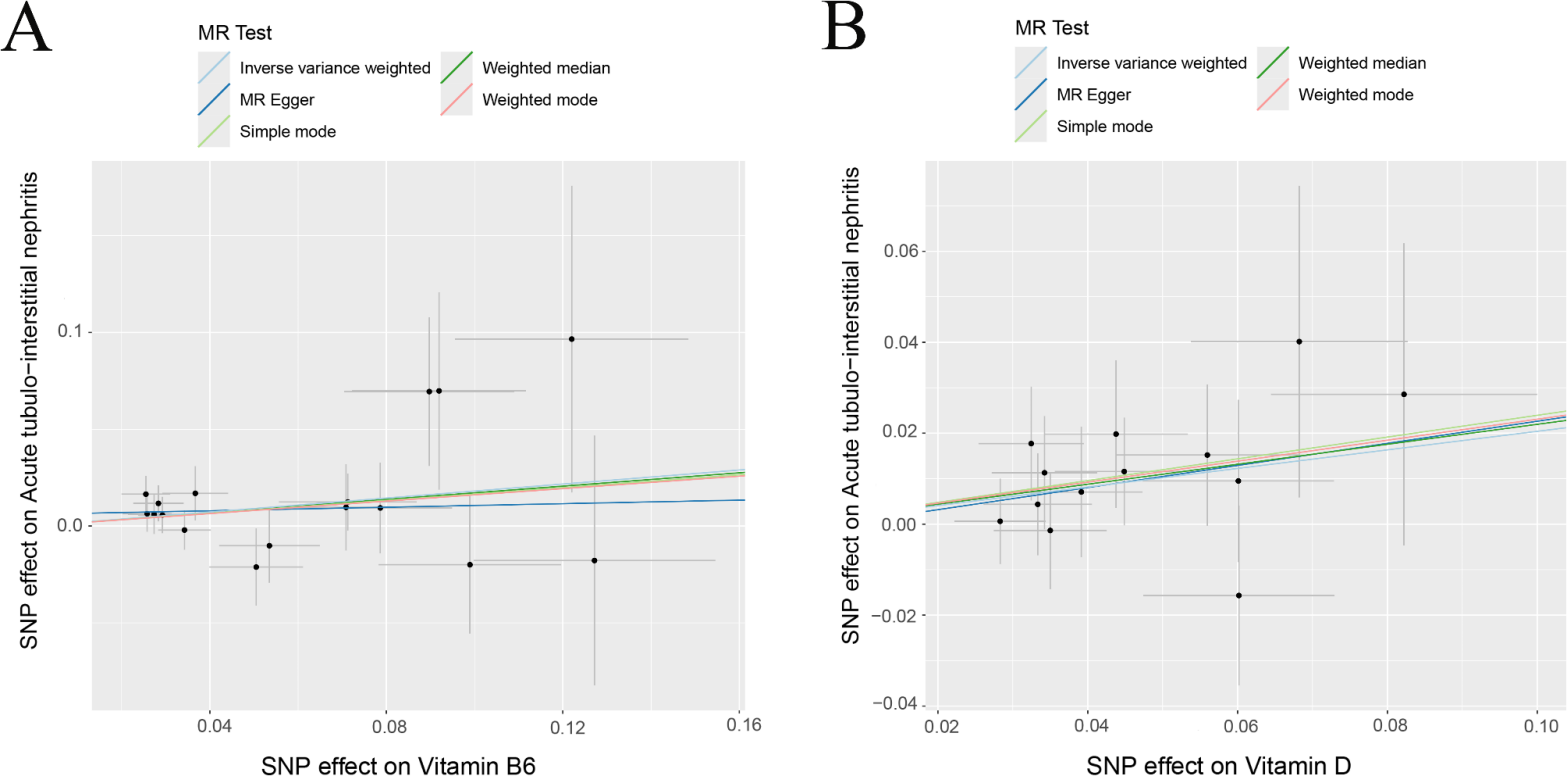
Scatter plot of the causal relationship between diseases. (**A**) Vitamin B6 with acute tubulointerstitial nephritis. (**B**) Vitamin D with acute tubulointerstitial nephritis

**Fig. 2:**
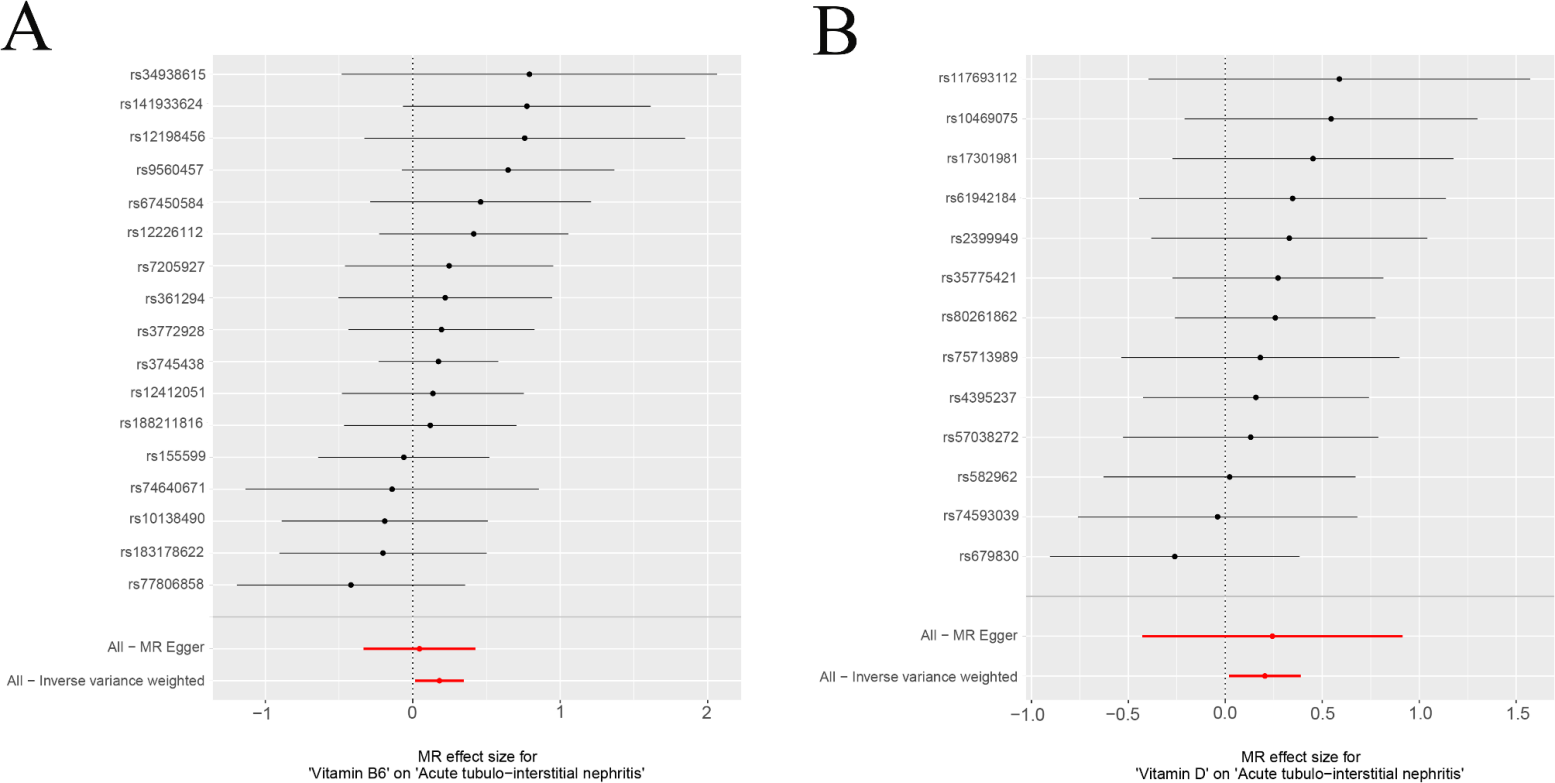
Forest plot of the effect size of each SNP. (**A**) Vitamin B6 with acute tubulointerstitial nephritis. (**B**) Vitamin D with acute tubulointerstitial nephritis

**Fig. 3:**
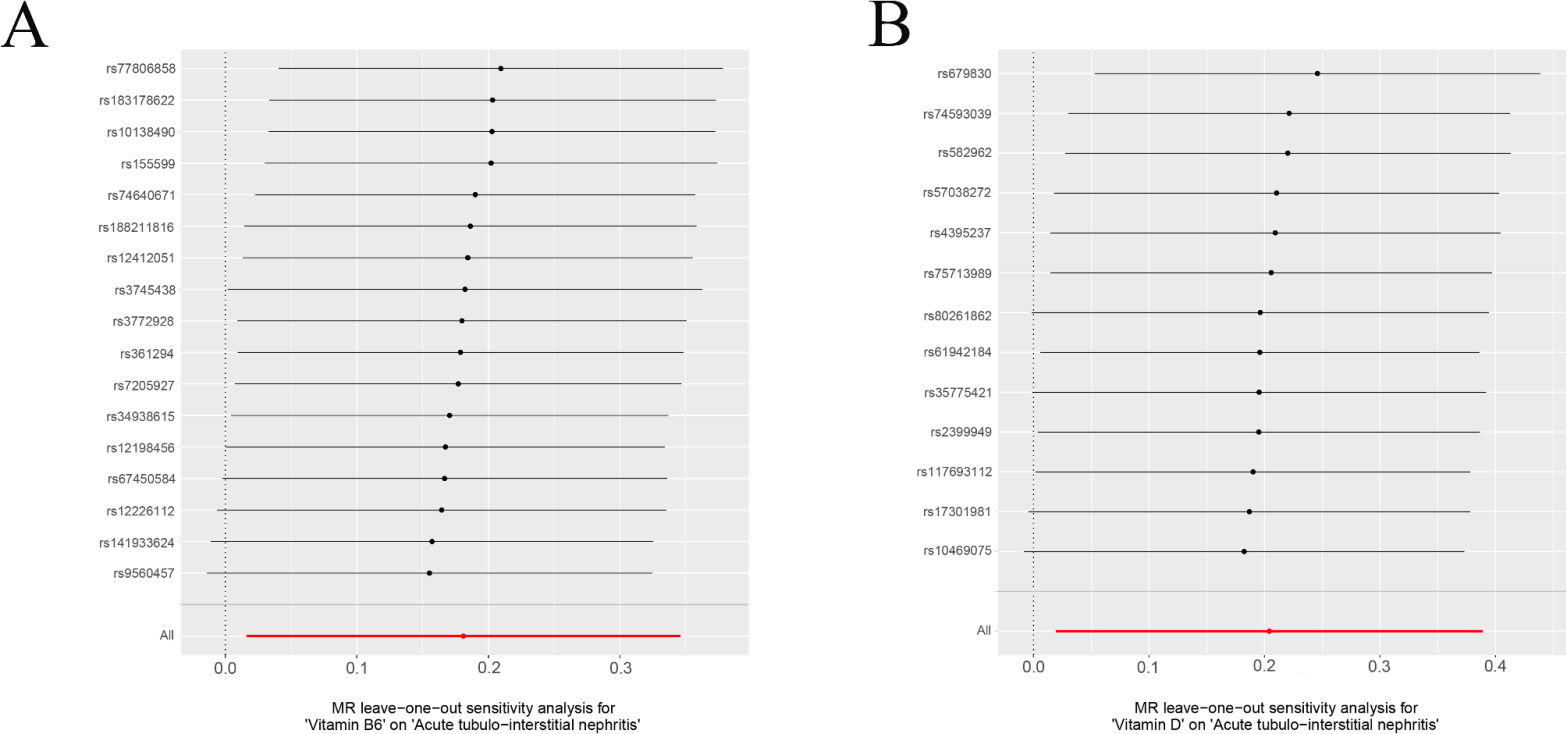
MR leave-one‐out sensitivity analysis. (**A**) Vitamin B6 with acute tubulointerstitial nephritis. (**B**) Vitamin D with acute tubulointerstitial nephritis

The MR Egger’s intercept test and the MR-PRESSO method were used to test for horizontal pleiotropy. *p* > 0.05 indicated that the genetic instrumental variables for the fourteen minerals and vitamins required by the human body were not horizontally pleiotropic for the GWAS of acute tubulointerstitial nephritis. *p* > 0.05 indicated that there was no heterogeneity in the MR analysis as detected by Cochran and Rucker’s Q statistic (Table 1). Among the fourteen minerals and vitamins required by the human body, vitamin B6 and vitamin D were genetically causal for acute tubulointerstitial nephritis in a two-sample Mendelian analysis. Results of IVW analysis of vitamin B6 for acute tubulointerstitial nephritis (β = 0.181; *P* = 0.032; OR = 1.198; 95% CI: 1.016–1.413) (Table 2). The results of IVW analysis of vitamin D for acute tubulointerstitial nephritis (β = 0.204; *P* = 0.030; OR = 1.226; 95% CI: 1.019–1.476)(Table 2). Each SNP of vitamin B6 versus vitamin D was shown by MR analysis to have an effect on acute tubulointerstitial nephritis (Fig. 1). This was similarly illustrated for the individual SNP effect value analysis (Fig. 2). In addition, in leave-one-out sensitivity analyses, when removing SNPs for any fourteen minerals and vitamins required by the human body, respectively, the final results received no effect (Fig. 3). In addition, our multi-sample Mendelian analysis of vitamin B6 versus vitamin D for acute tubulointerstitial nephritis found that, excluding the effect of vitamin D, vitamin B6 produced independent genetic causality for acute tubulointerstitial nephritis. The results of their IVW analysis (*P* = 0.010; 95% CI: 0.021–0.159). Thus, our analysis suggests that there is a causal relationship at the genetic level of vitamin B6 for acute tubulointerstitial nephritis with a negative correlation.

**Table 1:** Tests of Pleiotropy and heterogeneity of genetic instrumental variants

Pleiotropy test (vitamin B6 with acute tubulointerstitial nephritis genetic IVs)				Heterogeneity test (vitamin B6 with acute tubulointerstitial nephritis genetic IVs)						
**mr_egger**			**PRESSO**	**mr_egger**			**IVW**			
**Intercept**	**SE**	**P**	**P**	**Q**	**Q_df**	**P**	**Q**	**Q_df**	**P**	
0.006	0.008	0.450	0.764	11.623	15	0.707	12.225	16	0.728	
**Pleiotropy test (vitamin D with acute tubulointerstitial nephritis genetic IVs)**				**Heterogeneity test (vitamin D with acute tubulointerstitial nephritis genetic IVs)**						
**mr_egger**			**PRESSO**	**mr_egger**			**IVW**			
**Intercept**	**SE**	**P**	**P**	**Q**	**Q_df**	**P**	**Q**	**Q_df**	**P**	
-0.002	0.014	0.908	0.950	4.969	11	0.933	4.983	12	0.959	

*Abbreviations*: GWAS, genome-wide association study; IVW, inverse variance weighted; MR, Mendelian randomization; SE, standard error

*p* > 0.05 represents no significant pleiotropy and heterogeneity

**Table 2:** The mutual causal association of exposure and outcome

Method	N	β	SE	P	OR	95% CI
**Vitamin B6 with acute tubulointerstitial nephritis**						
mr_egger	17	0.046	0.194	0.817	1.047	0.716–1.530
Weighted median	17	0.172	0.115	0.135	1.188	0.948–1.488
IVW	17	0.181	0.084	0.032	1.198	1.016–1.413
Simple mode	17	0.166	0.185	0.384	1.180	0.821–1.695
Weighted mode	17	0.161	0.153	0.308	1.175	0.871–1.585
**Vitamin D with acute tubulointerstitial nephritis**						
mr_egger	13	0.243	0.343	0.493	1.275	0.651–2.496
Weighted median	13	0.220	0.123	0.075	1.245	0.978–1.585
IVW	13	0.204	0.094	0.030	1.226	1.019–1.476
Simple mode	13	0.239	0.195	0.244	1.270	0.866–1.863
Weighted mode	13	0.231	0.175	0.212	1.260	0.893–1.776

*Abbreviations*: IVW, inverse variance weighted; MR, Mendelian randomization; N, number of single-nucleotide polymorphism; β, the size of the obesity effect allele’s regression coefficient; SE, standard error; OR, odds ratio; 95%CI, 95% confidence interval. *p* < 0.05 indicates a causal relationship between exposure and outcome

### There is no genetic causality between fourteen essential minerals and vitamins and chronic tubulointerstitial nephritis

Using the above method to detect horizontal polytropy and heterogeneity in fourteen minerals and vitamins required by the human body for chronic tubulointerstitial nephritis, *p* > 0.05 indicated that there was no horizontal polytropy and heterogeneity in the GWAS of the instrumental variables for the outcome variables (Supplementary Material 2). There was no genetic causality in fourteen human-required minerals and vitamins for chronic tubulointerstitial nephritis in a two-sample Mendelian analysis with IVW analysis (*p* > 0.05) (Supplementary Material 3).

## Discussion

Immune-mediated nephritis is now recognized as the main cause of tubulointerstitial nephritis [25]. In addition, analysis of urine composition and renal biopsy are currently commonly used diagnostic tools. Based on this, the diagnosis and treatment of tubulointerstitial nephritis lack certain sensitivity and specificity [26]. Therefore, we need to explore the etiology of the disease at a deeper level and find more reliable diagnostic biomarkers. Therefore, there is a need for a broader study of this disease at the genetic level. Some single nucleotide polymorphisms in HLA or cytokine genes have been reported to increase susceptibility to acute tubulointerstitial nephritis [27]. Although a small number of studies have reported some association with tubulointerstitial nephritis at the genetic level, However, the kidney serves as an important excretory organ in the body. Nutrient absorption and excretion also appear to be particularly important for renal interactions. Therefore, we conjecture whether a certain nutrient is associated with tubulointerstitial nephritis at the gene level. This provides favorable evidence for further investigation of tubulointerstitial nephritis prevention, diagnosis, and treatment. Therefore, in this study, we used Mendelian randomization to analyze whether there is an effect of fourteen essential minerals and vitamins on tubulointerstitial nephritis from a genetic perspective. We interestingly found that among all the minerals and vitamins, vitamin B6 had a protective effect against acute tubulointerstitial nephritis.

The relationship between B vitamins and the kidneys is inextricably linked. One of the main roles of B vitamins is to participate in energy metabolism and protein synthesis, which is particularly important for people with kidney disease. Impaired kidney function may lead to metabolic abnormalities, and proper intake of B vitamins can help maintain metabolic balance [28]. Cardiovascular disease is a common complication in patients with kidney disease. Vitamin B6 and vitamin B12 can reduce the risk of cardiovascular disease by lowering homocysteine levels in the blood [29]. In patients with kidney disease, anemia may occur due to decreased kidney function. Proper intake of B vitamins can help improve anemia [30]. Vitamin B12 and folic acid are essential nutrients for red blood cell production. Vitamin B6, one of the B vitamins, is a water-soluble vitamin that can be obtained in a variety of foods. It can act as a coenzyme for various metabolic functions of proteins, carbohydrates, and lipids. Currently, in clinical practice, vitamin B6 is mainly used in connection with dermatologic and neurologic disorders. Vitamin B6 deficiency may lead to symptoms such as dry skin, rashes, and xerostomia. Vitamin B6 plays a role in the synthesis of neurotransmitters, such as serotonin and dopamine, which directly affect mood, sleep, and cognitive function [31]. In addition, vitamin B6 intake and supplementation have been found to improve some immune functions in vitamin B6-deficient individuals and experimental animals [32]. Vitamin B6 deficiency suppresses the function of T-lymphocytes and reduces the body’s resistance to viruses and bacteria. It may be that vitamin B6 acts as a cofactor in the pathway of metabolites with immunomodulatory effects [33]. Considering the progression of inflammatory and oxidative stress processes associated with the impairment of renal function and the development of renal histopathology, factors such as vitamin B deficiency may contribute to the development of chronic kidney disease.

Thus, vitamin B6, which has immunomodulatory properties, appears to be associated with immune-mediated tubulointerstitial nephritis. Although there may be a complex pathologic and physiologic relationship between the two, There are no basic or clinical studies that have illustrated the relationship. Only studies have explored the relationship between vitamin B6 and other diseases of the kidney. One study showed that chronic vitamin B6 deficiency caused kidney stones in rats [34]. In addition, one cohort study showed that circulating vitamin B6 may provide additional prognostic information on tumor staging in patients with kidney cancer and was negatively associated with kidney cancer risk and kidney cancer prognosis [35]. Vitamin deficiencies are common in chronic kidney disease. A cohort study with a 12-year follow-up found that a high dietary intake of vitamin B6 (≥ 1.6 mg/day) was associated with an increased risk of chronic kidney disease stage 3B and was higher compared with the recommended level of intake [36]. Vitamin B6 deficiency was found in studies of long-term adverse outcomes of renal transplantation and was independently associated with an increased risk of death from renal transplantation and was independently associated with an increased risk of death from renal transplantation [37]. Vitamin B6 deficiency may appear to have adverse effects on the kidney and has been studied in many diseases of the kidney. However, no studies have been done before this to illustrate the effect of vitamin B6 on tubulointerstitial nephritis. Therefore, the present study has found a protective effect of vitamin B6 in acute tubulointerstitial nephritis.

## Conclusion

We genetically predicted the possible influence of minerals and vitamins on acute and chronic tubulointerstitial nephritis. Vitamin B6 deficiency in vivo was found to adversely affect acute tubulointerstitial nephritis. Thus, different minerals and vitamins have different effects on different forms of tubulointerstitial nephritis. This puts a demand for more precise dietary nutritional intake. Currently, with the continuous development of nutrigenomics, individual and disease differences demand that we treat and prevent diseases from a genetically inherited perspective. This has led to the need to continually incorporate the therapeutic paradigm of different nutrients influencing disease outcomes through different genetic differences into clinical applications. The present study is limited to exploring the relationship that exists between nutrients and tubulointerstitial nephritis, starting with minerals and vitamins only. This requires us to start from the perspective of more nutrients in the future, and through more mechanistic studies and clinical explorations, we need to take a closer look at the mechanisms affecting the development of tubulointerstitial nephritis, which can help in the prevention, diagnosis, and treatment of the disease.

## Data Availability

No datasets were generated or analysed during the current study.

## Abbreviations

MR: Mendelian randomization
IVW: Inverse variance weighting
GWAS: Genome-wide association study
IVs: Instrumental variables
SNPs: Single nucleotide polymorphisms
EA: Effect allele
NEA: Non-effect allele
EAF: Effect allele frequency
